# Pavement ant extract is a chemotaxis repellent for C. elegans

**DOI:** 10.17912/micropub.biology.001146

**Published:** 2024-03-25

**Authors:** Jayela S. Lopez, Saif Ali, Malcom Asher, Christina A. Benjamin, Ryan T. Brennan, Mai Ly T. Burke, Joseph M. Civantos, Emilia A. DeJesus, Ana Geller, Michaela Y. Guo, Sophia K. Haase Cox, Julia M. Johannsen, Joshua S. J. Kang, Harrison B. Konsker, Benjamin C. Liu, Kylie G. Oakes, Hannah I. Park, Diego R. Perez, Amin M. Sajjadian, Madeleine Torio Salem, Justine Sato, Amanda I. Zeng, Bryan H. Juarez, Mabel Gonzalez, Griselda Morales, Nicole Bradon, Katherine Fiocca, Mila M. Pamplona Barbosa, Lauren A. O'Connell

**Affiliations:** 1 BIO161 Organismal Biology Lab, Stanford University, Stanford, California, United States; 2 Department of Biology, Stanford University, Stanford, California, United States

## Abstract

Ant behavior relies on a collection of natural products, from following trail pheromones during foraging to warding off potential predators. How nervous systems sense these compounds to initiate a behavioral response remains unclear. Here, we used *Caenorhabditis elegans* chemotaxis assays to investigate how ant compounds are detected by heterospecific nervous systems. We found that *C. elegans* avoid extracts of the pavement ant ( *Tetramorium immigrans* ) and either *osm-9* or *tax-4* ion channels are required for this response. These experiments were conducted in an undergraduate laboratory course, demonstrating that new insights into interspecies interactions can be generated through genuine research experiences in a classroom setting.

**Figure 1. Pavement ant ( Tetramorium immigrans ) extract induces an osm-9 or tax-4 dependent repulsive chemotaxis response in C. elegans .**
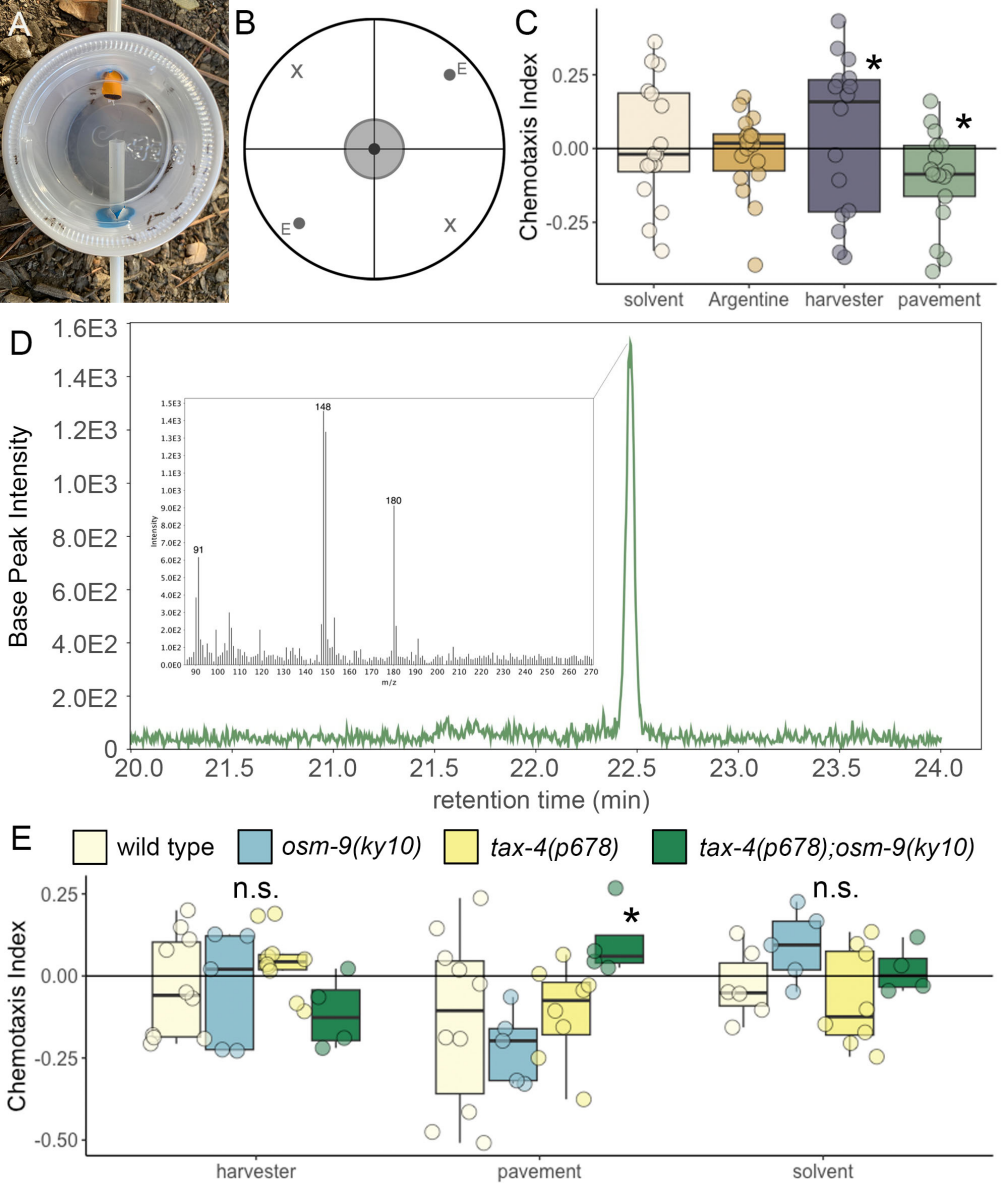
**(A)** Students constructed simple aspirators to collect ants. Photo from Alfonso et al., 2023. **(B)** Chemotaxis assays were performed on circular plates divided into quadrants. Worms were placed in the center and exposed to ant extracts (E) and solvent (X). **(C)** The chemotaxis response of wild-type worms (PD1074) was tested in response to Argentine ant ( *Linepithemma humile,* orange), Western harvester ant ( *Pogonomyrmex occidentalis,* purple), or pavement ant ( *T. immigrans,* green) extracts. Wild-type PD1074 worms were attracted to harvester ant extracts and repulsed by pavement ant extract compared to solvent (white). **(D)** Detection of trail pheromone methyl 2-methoxy-6-methylbenzoate (MMMB) at 22.5 min. Base peak chromatograms from fragments m/z 148-149 were extracted and representative ions m/z 91, 148, 149 and 180 were detailed in the corresponding mass spectrum (insert). **(E)** The chemotaxis response of wild type worms (PD1074, white), *osm-9(ky10)* knockout worms (blue), *tax-4(p678)* knockout worms (yellow), and *tax-4(p678);osm-9(ky10)* double mutants (green) were tested in response to Western harvester and pavement ant extracts. While worm strains did not respond to Western harvester extract or solvent, the repulsive effect of pavement ant extract was diminished in the *tax-4(p678);osm-9(ky10)* double mutants. Asterisks indicate significant differences; n.s. (not significant) indicates comparisons with a p-value above 0.05.

## Description

Ant social behavior is reliant on a collection of pheromones that allow them to exhibit complex social organization, from collecting food to warding off predators (Berridge, 1985; Jackson and Ratnieks, 2006) . However, knowledge of the neurological mechanisms that allow these chemicals to influence behavior remains limited (Adams et al., 2020) . This study aims to use *Caenorhabditis elegans* and chemotaxis assays to identify genes and neurons responsible for eliciting responses to ant-derived compounds. *C. elegans* is a useful model for exploring these relationships because of its well studied nervous system and genetic resources (Sengupta and Samuel, 2009) . Moreover, the *C. elegans* chemosensory system is well understood, laying a foundation for probing the neural mechanisms underlying interspecies interactions (Colbert et al., 1997; Komatsu et al., 1996) *.* With these tools, our main goal was to use *C. elegans* as an experimental system to identify neuronal mechanisms responsible for ant compound detection.

To examine the influence of ant-derived compounds on *C. elegans* chemotaxis behavior, we tested the response of wild-type ( PD1074 ) worms (Yoshimura et al., 2019) to extracts of three different ant species ( Figure 1A -C): the invasive Argentine ant ( *Linepithema humile* ), the native Western harvester ant ( *Pogonomyrmex occidentalis* ), and the invasive pavement ant ( *Tetramorium immigrans* ). Wild-type worms varied in their response to compounds from different ant species (GLMM: X ^2^ (3) = 860.14, p < 0.001). While worms were indifferent to Argentine ant extracts (z = 1.436, ratio ± SE = 1.018 +/- 0.013, p _adj_ = 0.151), they were attracted to Western harvester ant extracts (z = 21.310, ratio ± SE = 1.278 +/- 0.015, p _adj_ < 0.001) and avoided pavement ant extracts (z = -4.604, ratio ± SE = 0.947 +/- 0.011, p _adj_ < 0.001). This indifference to Argentine extracts contrasts with prior findings showing *C. elegans* are repulsed by these compounds (Alfonso et al., 2023) . This discrepancy could be due to natural variation in ant chemical signatures, as there is high variability in Argentine ant chemical profiles within and between colonies (Salado et al., 2023) . Alternatively, this difference could be based on the concentration of the compounds in the extracts, as the concentration of chemical stimuli can influence *C. elegans* chemotaxis outcomes (Yoshida et al., 2012) . Compared to Argentine ants, limited work has been done on the chemistry of pavement ants, although a trail pheromone has been identified as methyl 2-methoxy-6-methylbenzoate (Chalissery et al., 2022) . This compound was detected in the pavement ant extract using gas chromatography / mass spectrometry ( Figure 1D ), although we do not know if this specific compound is responsible for avoidance behavior in *C. elegans* . Future work identifying whether this or other pavement ant compounds are responsible for repulsing *C. elegans* would be valuable to better understand the chemistry of this invasive ant species.

We next asked whether chemosensory neurons were required for these differential chemotaxis responses to specific ant extracts. Chemosensory signaling in *C. elegans* neurons can be inactivated by removing specific ion channels like OSM-9 (Colbert et al., 1997) and TAX-4 (Dusenbery et al., 1975; Komatsu et al., 1996) . These ion channels are each expressed in mostly-distinct subsets of chemosensory neurons (Fryer et al., 2023) . We tested the responses of *osm-9 ( ky10 )* null mutants (Colbert et al., 1997) , *tax-4 ( p678 )* null mutants (Komatsu et al., 1996) , *tax-4 ( p678 ); osm-9 ( ky10 )* double mutants (Fryer et al., 2023) , and wild-type worms ( PD1074 ) to Western harvester and pavement ant extracts ( Figure 1E ). We found that combinations of ant extracts and worm strain influenced chemotaxis behavior (GLMM compound*strain: X ^2^ (6) = 17.450, p = 0.008). Worm strains did not differ in their response to Western harvester ant extracts or to solvent. However, double mutant responses to pavement compounds were significantly different compared to both single *osm-9 ( ky10 )* and *tax-4 ( p678 )* null mutants and wild type (double mutant versus wild type: t(66) = 2.765, p _adj_ = 0.022; double mutant versus *tax-4* null mutant: t(66) = 2.400, p _adj_ = 0.038; double mutant versus *osm-9* null mutant: t(66) = 3.117, p _adj_ = 0.016). Wild type, single *tax-4* null mutants, and *osm-9* null mutants did not differ in their responses to pavement ant compounds (p > 0.420). These results suggest that *C. elegans* repulsive responses to pavement ant extracts require either *osm-9* or *tax-4* . Repulsion by Argentine ant extracts is dependent on *osm-9* , but not *tax-4* (Alfonso et al., 2023) , suggesting that the chemical compositions of different ant species influence the *C. elegans* chemosensory system via multiple mechanisms. Future work with additional mutants or ant extract fractions would allow us to better understand the underlying compounds, signaling pathways, and odorant receptors responsible for this chemotaxis response.

In summary, we have shown that the extracts of the pavement ant is a repellent for *C. elegans* and that this response requires either *osm-9* or *tax-4* , suggesting there are multiple different neurons at play in generating the chemosensory response or a subset of chemosensory neurons requires both genes to function. Future studies will include additional mutant and divergent worm strains and more ant species. Overall this work demonstrates that pavement ant compounds can influence heterospecific chemosensory systems and extracts from different ant species target nervous systems by different mechanisms. Moreover, *C. elegans* provides a genetically tractable system for studying the genetic and neural mechanisms mediating these interspecies interactions within an undergraduate classroom experience.

## Methods

*Worm strains*

Wild-type and mutant worm strains were obtained from the Caenorhabditis Genetics Center (CGC) at the University of Minnesota or from the lab of Miriam Goodman at Stanford University. Animals were maintained in 20°C incubators and nematodes were synchronized by bleaching adults to obtain eggs. Roughly 300-500 eggs were pipetted onto Nematode Growth Media plates spread with OP50 *E. coli* . NGM plates were made as described (Stiernagle, 2006) . Hatched eggs were kept at 20°C for roughly 3 days when the population reached a young adult stage and were used for chemotaxis assays.

*Ant extracts*

The Argentine ant ( *Linepithema humile)* were collected on the Stanford University campus while the Western harvester ant ( *Pogonomyrmex occidentalis* ) and the pavement ant ( *Tetramorium immigrans* ) were obtained from a commercial supplier (Stateside Ants, USA). For field collection of ants, students built aspirators using plastic ketchup cups, plastic straws, putty, and strips of mesh. Ants were incubated at -80°C for 15 minutes and then sorted from debris into glass vials of methanol (1:1 methanol:ant volume). After incubating these vials for 24 hours at -80°C, the methanol was moved to a new glass vial, leaving the ants behind. Methanol samples intended for chemotaxis assays containing ant compounds were then evaporated under a constant flow of nitrogen gas to dryness. Evaporated ant extracts were then resuspended in 1:1 dimethyl sulfoxide:ant volume (DMSO). The remaining ant samples stored in methanol were retained at -80°C until chromatographic analyses were performed.

*Chemotaxis Assays*

Undergraduate students in a laboratory course performed chemotaxis assays while unaware of compound or strain being tested until data was submitted to the instructor. Chemotaxis plates [5mM KPO _4_ (pH 6), 1mM CaCl _2_ , 1mM MgSO _4_ , 2% agar] were divided into four quadrants ( Figure 1A ). Compounds (5 μL, ant extract, concentration unknown) were placed on dots located in two non-adjacent quadrants (E, experimental) while 5 μL of DMSO (solvent) was placed on X marks in the other two non-adjacent quadrants. Plates were then incubated for 30 minutes to allow for a chemical gradient to be established. During this incubation period, worms were removed from OP50 plates and washed three times with Chemotaxis Assay Buffer [5mM KPO _4_ (pH 6), 1mM CaCl _2_ , 1mM MgSO _4_ ]. Following compound incubation, 2 μL 0.5 M sodium azide solution was applied to each of the quadrant spots to paralyze the worms at those locations. Then, roughly 100 worms were placed in the center of each plate. Worms were allowed to roam the plate for 30 min and then were counted manually under a dissecting microscope using a tally counter. Worms in the center dot of overlapping quadrants were not counted to avoid including dead worms in the dataset. Twenty-two students conducted all the experiments, and each assay was replicated 4-22 times, depending on the assay.

*Gas Chromatography/Mass Spectroscopy Analysis*

Ant samples were analyzed by gas chromatography coupled to mass spectrometry (GC/MS) on a system consisting of a GC-2030 NEXIS, a single quadrupole mass spectrometer (QP-2020), and an autosampler SPL AOC-20i (Shimadzu, Nakagyo-ku, Kyoto, Japan). From the methanol ant extract, 1uL was injected in splitless mode at 250°C. The separation was achieved on a HP-5MS capillary GC column (30 m × 0.25 mm × 0.25 μm, Agilent, Palo Alto, CA, USA) at a flow rate of 1.0 mL/min. The temperature program started at 40°C for 3 minutes and then was increased to 100°C at rate of 6°C/min, then to 200°C at 4°C/min, and finally was increased to 300°C at rate of 20°C/min, and maintained at this temperature for 3 min. The ion source and the interface temperature were set at 150°C and 230°C, respectively. The mass spectrometer was operated in full scan mode using a mass range of 40-500 m/z and performing a scan every 0.2s. The GC/MS file was converted to .CDF format and opened in MZmine3 (Schmid et al., 2023) to create panel D, showing the chromatogram corresponding to the trail pheromone methyl 2-methoxy-6-methylbenzoate (MMMB) (Chalissery et al., 2022) , filtering the base peak intensity ions m/z 148 and 149 and retention time between 20-24 min. Putative annotation of this compound was performed using GCMS Postrun Analysis software (Shimadzu) employing NIST 14 and Wiley databases. Mass spectrometry data are provided as supplementary extended files.

*Data Analysis*

The Chemotaxis Index (CI) was calculated for each plate, where CI = (Number of worms in the two experimental quadrants – Number of worms in the two solvent quadrants) / Total number of worms on the entire plate. Plates were removed from the data set prior to analysis if there were less than 40 worms on the plate or if a student noted a technical error in the plate setup, such as mistakes in pipetting compounds or worms onto the appropriate locations.

Data analysis and visualization were performed in R (version 4.3.0) in RStudio (version 2023.03.1+446). We used the glmmTMB package (version 1.1.7; (Brooks et al., 2017) ) for statistical analyses using generalized linear mixed models and followed each model with the Anova.glmmTMB function for reported statistical values. Appropriate model diagnostics were confirmed with the DHARMa package (version 0.4.6; (Hartig, n.d.)). For the initial ant screen, the number of worms in the experimental quadrants was used as the dependent variable and compound as the independent variable with residuals having a negative binomial distribution. Student was used as a random effect and the total numbers of worms per plate were used as weights in the model. For the experiment with different worm strains, the model used in the previous experiment led to residual heteroscedasticity issues. To account for this, we used the number of worms in the experimental quadrants divided by the total number of worms on the plate as the dependent variable while compound, worm strain, and their interaction were independent variables with student as a random effect and residuals having a Gaussian distribution. *Post hoc* analyses were performed using either emmeans (version 1.8.6) or grafify (version 4.0.0) packages with false discovery rate (fdr) adjustment of p-values to account for multiple testing. Pairwise *post hoc* tests were run for the mutant strain experiment while the initial ant extract screen was only compared to the control group. Boxplots were generated using the ggplot2 (version 3.4.3) package.

*Classroom pedagogy*

We conducted the experiments described here across three laboratory sessions. These sessions were preceded with one training session where students learned how to conduct chemotaxis assays using a known attractant (isoamyl alcohol) and repellant (carvone) (Ellington et al., 2020) . One laboratory session involved collecting ants on campus using aspirators constructed by students. Two other laboratory sessions involved worm chemotaxis assays. Weekly homework included reading relevant literature, data analysis and visualization, and writing the results and interpretations. Assignments were marked as complete/incomplete with detailed feedback at each stage. The final project was to write this journal-style article. Each student wrote an individual article, received detailed instructor feedback, and then submitted the final version. The instructor then merged these individual articles into a single document to create this manuscript. All students edited the final manuscript and approved of its submission.

## Reagents

**Table:** 

Strain Name	Genotype	Source
PD1074	Wild type	Caenorhabditis Genetics Center (CGC) at the University of Minnesota
PR678	*tax-4 ( p678 )* III	Caenorhabditis Genetics Center (CGC) at the University of Minnesota
CX10	*osm-9 ( ky10 )* IV	Caenorhabditis Genetics Center (CGC) at the University of Minnesota
GN1077	*tax-4 ( p678 )* III *; osm-9 ( ky10 )* IV	Miriam Goodman Lab at Stanford University

## Extended Data

Description: pavement ant extract gcms. Resource Type: Dataset. DOI: 10.22002/1nx1x-8jp79

Description: gcms blank control. Resource Type: Dataset. DOI: 10.22002/vb73p-3gw37

Description: gcms alkane control. Resource Type: Dataset. DOI: 10.22002/yeazy-5c026

Description: read me file. Resource Type: Dataset. DOI: 10.22002/xfxeb-xgn94
